# Deciphering the Broad Antimicrobial Activity of *Melaleuca alternifolia* Tea Tree Oil by Combining Experimental and Computational Investigations

**DOI:** 10.3390/ijms241512432

**Published:** 2023-08-04

**Authors:** Federico Iacovelli, Alice Romeo, Patrizio Lattanzio, Serena Ammendola, Andrea Battistoni, Simone La Frazia, Giulia Vindigni, Valeria Unida, Silvia Biocca, Roberta Gaziano, Maurizio Divizia, Mattia Falconi

**Affiliations:** 1 Department of Biology, University of Rome Tor Vergata, Via della Ricerca Scientifica 1, 00133 Rome, Italy; federico.iacovelli@uniroma2.it (F.I.); alice.romeo@uniroma2.it (A.R.); patriziolattanzioo@gmail.com (P.L.); serena.ammendola@uniroma2.it (S.A.); andrea.battistoni@uniroma2.it (A.B.); simone.la.frazia@uniroma2.it (S.L.F.); 2 Department of Systems Medicine, University of Rome Tor Vergata, Via Montpellier 1, 00133 Rome, Italy; giuliavindy@hotmail.it (G.V.); valeria.unida@gmail.com (V.U.); biocca@med.uniroma2.it (S.B.); 3 Microbiology Section, Department of Experimental Medicine, University of Rome Tor Vergata, Via Montpellier, 1–00133 Rome, Italy; roberta.gaziano@uniroma2.it; 4 Department of Biomedicine and Prevention, University of Tor Vergata, 00133 Rome, Italy; divizia@uniroma2.it

**Keywords:** tea tree oil, molecular dynamics, molecular docking, eukaryotic cells viability, antiviral activity, antimicrobial activity, antifungal activity, membrane partitioning, viral capsid binders

## Abstract

Tea Tree Oil (TTO) is an essential oil obtained from the distillation of *Melaleuca alternifolia* leaves and branches. Due to its beneficial properties, TTO is widely used as an active ingredient in antimicrobial preparations for topical use or in cosmetic products and contains about 100 different compounds, with terpinen-4-ol, γ-terpinene and 1,8-cineole (or eucalyptol) being the molecules most responsible for its biological activities. In this work, the antimicrobial activity of whole TTO and these three major components was evaluated in vitro against fungi, bacteria and viruses. Molecular dynamics simulations were carried out on a bacterial membrane model and a Coxsackievirus B4 viral capsid, to propose an atomistic explanation of their mechanism of action. The obtained results indicate that the strong antimicrobial activity of TTO is attributable to the induction of an altered membrane functionality, mediated by the incorporation of its components within the lipid bilayer, and to a possible ability of the compounds to bind and alter the structural properties of the viral capsid.

## 1. Introduction

Essential oils are a complex mixture of volatile secondary metabolites, responsible for the distinctive smell or taste of plants, which are extensively used in cosmetics or food flavoring. They have been used for centuries in folk medicine, accounting for many of the beneficial effects exerted by medicinal plants, and a lot of interest has been directed toward their use as therapeutic alternatives for different pathological conditions [1]. Tea Tree Oil (TTO) is a volatile essential oil mainly derived from water vapor distillation of leaves and terminal branches of *Melaleuca alternifolia* (Myrtaceae), a small tree endemic to Australia [1,2,3]. It was originally exploited by aboriginal peoples of the Australian mainland as an antiseptic and herbal medicine to treat cough, cold or skin disease, and its medicinal properties were first reported in 1923 [2]. Several papers described its broad-spectrum antibacterial, antifungal, antiprotozoal and antiviral activities, and nowadays TTO is incorporated in different dermatological and oral hygiene products [1,4]. Clinical studies demonstrated its efficacy in the decolonization of methicillin-resistant *S. aureus* and in treating skin and mucosal infections, including acne, seborrheic dermatitis, cold sores, oral candidiasis and chronic gingivitis, representing an effective natural approach for the treatment of infectious diseases [2,4,5,6]. The possible use of TTO in handwash formulations for healthcare settings has also been suggested [7]. In addition to its antimicrobial properties, TTO can also accelerate the wound healing process and exhibits anti-inflammatory and antitumoral activities [4,8,9]. TTO contains about 100 different components, including aromatic terpene hydrocarbons, sesquiterpenes and their associated alcohols [10]. Different plant chemotypes and storage conditions result in variations in the chemical composition of commercially available oils [11,12]. 

Most promising antimicrobial properties were observed with the terpinen-4-ol TTO chemotype, characterized by 30–40% terpinen-4-ol content [1,12]. Indeed, most of its antimicrobial properties are generally attributed to terpinen-4-ol (also known as 4-carvomenthenol) [2,13], although it is supposed that TTO antimicrobial mechanism results from complex additive interactions between its different components [11]. Many studies showed that most clinically relevant bacteria are susceptible to low TTO concentrations, confirming its role as an effective bactericidal agent [2,11,14,15]. Antimycotic activities of TTO and its major components were also widely assessed on numerous species [2,16,17,18,19,20,21,22]. The antimicrobial activity mediated by essential oils and their components has been explained by the impairment of the structural and functional integrity of bacterial and fungal membranes. In fact, because of their lipophilic character, cyclic monoterpenes would preferentially partition from the aqueous phase into the lipid membrane environment, resulting in membrane expansion, increased fluidity and inhibition of membrane-embedded enzymes [2,23,24,25,26,27,28,29,30,31]. Biophysical and ultrastructural studies on simplified membrane models (Langmuir film) showed that TTO could interpose the lipid bilayer thanks to its affinity for the lipids’ hydrophobic tails, preferentially interacting with the less-ordered dipalmitoylphosphatidylcholine (DPPC) “sea”, and not altering the more ordered raft structure [32]. 

While substantial progress has been made in outlining TTO’s antibacterial and antifungal properties, little data are available on the mechanism of its antiviral and/or virucidal activity. Different studies described the efficacy of TTO or some of its components against the herpes simplex virus (HSV-1) [33,34,35], which seems to be primarily due to a direct effect of oil components on the extracellular virus particles. No significant plaque reduction could be detected with pre-treatment of cells before virus addition or post-treatment after viral absorption [34]. Accordingly, TTO is beneficial in treating recurrent herpes labialis [36]. TTO is also active against the influenza virus and different coronaviruses, inhibiting their entry into the host cells probably through the interaction of its components with the viral envelope, interfering with the biological activity of key viral structures involved in the fusion process [6,37,38,39,40,41,42]. On the other hand, non-enveloped viruses are more resistant than enveloped viruses to antiviral/virucidal treatment with essential oils (EOs), including TTO [40,41,43]. Concerning its toxicity, if correctly used and conserved, TTO can be considered safe [44,45]. Indeed, known adverse reactions are minor, dose dependent, self-limiting and occasional. Death due to TTO intoxication has never been reported in the literature, although it can be toxic if ingested at high doses, and topical and repeated use at high concentrations can cause dermatitis. 

In this work, TTO and its three major components (terpinen-4-ol, γ-terpinene and 1,8-cineole) were experimentally tested on eukaryotic cells, fungi, bacteria and viruses to identify a possible distinct activity of the whole oil and individual compounds. Following experimental results, classical molecular dynamics (MD) simulations were carried out to propose an atomistic explanation for the observed effects. The simulated systems include a bacterial membrane portion with an embedded peptidoglycan glycosyltransferase protein, in the presence and absence of the three evaluated TTO compounds. As a test bed, a virus capsid portion of Coxsackievirus B4 was also simulated in the presence of terpinen-4-ol molecules. The results here presented suggest that, like other essential oils, the TTO mixture performs its antimicrobial activity in a non-specific and concerted way. The mechanism of action of TTO could mainly be ascribed to an alteration of membrane function, induced by the insertion of its compounds within the lipid bilayer. Additionally, this oil may also disrupt the viral infection process by hindering the disassembly of non-enveloped viruses.

## 2. Results

### 2.1. Experimental Results

To evaluate the effect of TTO and its major components (terpinen-4-ol, γ-terpinene and 1,8-cineole) on different microorganisms, a series of experiments were carried out on cells, bacteria, fungi and non-enveloped viruses. The experimental results were then analyzed considering atomistic simulations outcomes to propose a mechanism of action for TTO and its major components. 

#### 2.1.1. Effect of TTO and Its Components on Bacteria 

**Figure 1:**
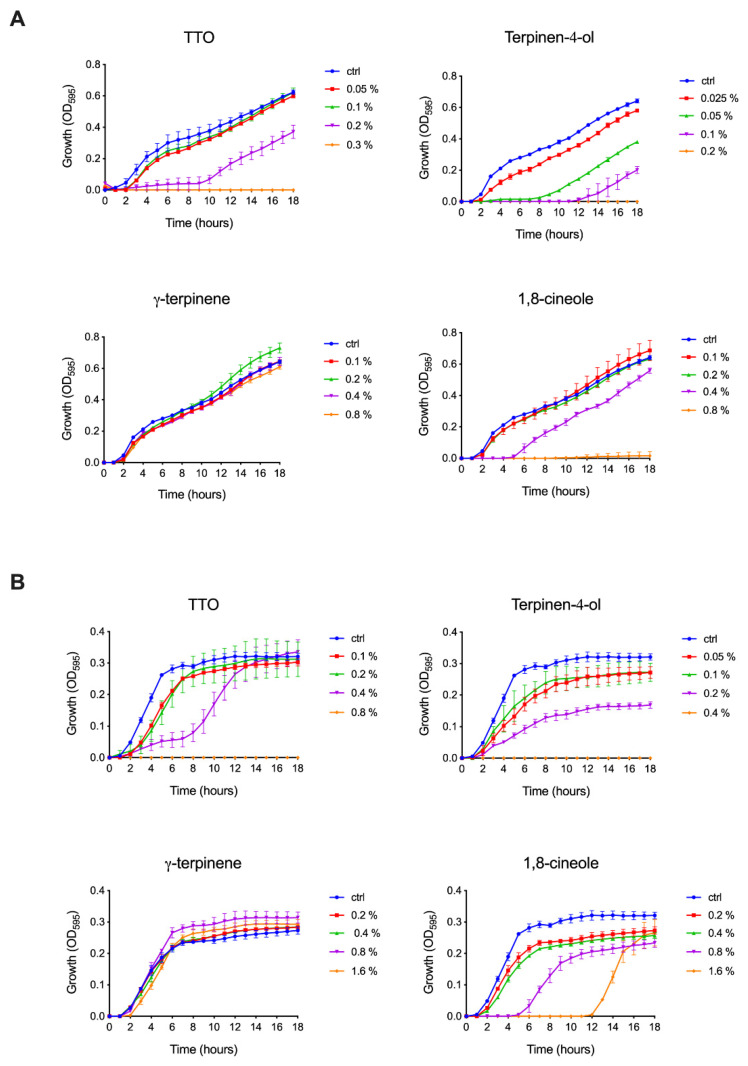
Effect of TTO, terpinen-4-ol, γ-terpinene and 1,8-cineole on (**A**) *S. typhimurium* and (**B**) *S. aureus* growth. Control samples were grown in LB medium without any supplementation (ctrl), while compounds were tested at different concentrations (*v*/*v*), as indicated in the legends. Each absorbance value (OD595) is the mean value of three replicates.

To better describe the effect of TTO and its components on bacteria, we selected a Gram-negative and a Gram-positive pathogenic species, i.e., *Salmonella enterica* ser. Typhimurium and *Staphylococcus aureus*, characterized by different cellular envelope morphologies. As a didermic species, *S. typhimurium* is characterized by a thin peptidoglycan layer surrounding the cytoplasmic membrane and an outer lipidic membrane rich in lipopolysaccharide, bearing several porins that allow the exchange of a broad range of molecules with the periplasmic space. Conversely, the *S. aureus* shows a monodermic structure consisting of a thick peptidoglycan layer positioned outside the membrane. The effect of TTO, terpinen-4-ol, γ-terpinene and 1,8-cineole on bacteria was analyzed by monitoring their growth curves in the presence of different concentrations of the compounds. As shown in Figure 1, both species are sensitive to TTO, even if at different dilutions. *S. typhimurium* was shown to be completely inhibited by 0.3% TTO, while a comparable effect was obtained with *S. aureus* only at 0.8% TTO. At lower TTO concentrations, in both *S. typhimurium* and *S. aureus* (at 0.2% and 0.4%, respectively), a noticeable slowdown in growth is observed in the first eight hours, followed by a subsequent recovery of doubling time to the level of control samples. Among the TTO components, γ-terpinene was found to be completely ineffective in both species. At the same time, terpinen-4-ol has the highest growth inhibitory effect (0.2% for *S. typhimurium* and from 0.4% for *S. aureus*). The effect of 1,8-cineole on the two species is quite different. In *S. typhimurium*, it causes an almost complete inhibition of growth from 0.8% and a five-hour growth retardation at 0.4%, while *S. aureus* can still grow at 1.6% even after a twelve-hour delay. MIC_100_ values calculated for TTO and its components against *S. typhimurium* and *S. aureus* are reported in Table 1.

#### 2.1.2. Effect of TTO and Its Components on Fungi

The antifungal efficacy of TTO and its main components was evaluated against the fluconazole-sensitive *C. albicans* strain ATCC 2091. As reported in Table 2, the MIC_50_ for fluconazole, used as a control, was 0.5 µg/mL. *C. albicans* strains were classified as susceptible or resistant to fluconazole with MIC values ≤2 µg/mL or >4 µg/mL, respectively, according to the breakpoints recommended by the CLSI [46]. The experiments confirm the antifungal activity of TTO and its components, as previously described by other authors [11,17,20]. The results in Table 2 show that TTO displayed a remarkable dose-dependent antifungal activity against *C. albicans* with a MIC_50_ at 0.125% and a MFC at 0.25%. Also, the 1,8-cineole, γ-terpinene and terpinen-4-ol, when tested individually, were able to effectively inhibit the fungal growth, showing equal MIC_50_ and MFC values (0.25% and 0.5%, respectively), although their antifungal efficacy was slightly lower (one-fold lower) than that of the total plant extract. These results suggest that TTO components might work in combination, improving the fungistatic and fungicidal properties of TTO against *C. albicans*.

#### 2.1.3. Cytotoxic Effect of TTO and Its Components

**Figure 2:**
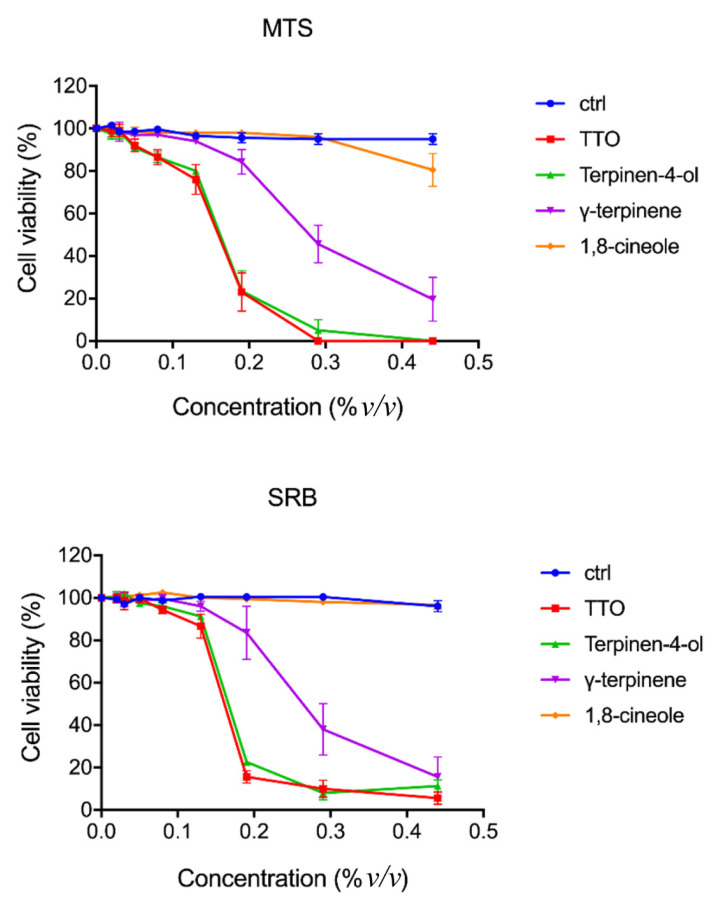
Cytotoxic effect of TTO and its components on BGM cells. Cell viability was assessed using MTS and SRB assays after 2 h treatment with TTO, terpinene-4-ol, γ-terpinene and 1,8-cineole at different concentrations, as indicated. The values are the mean ± S.E.M of three independent experiments, normalized to control.

The cytotoxic effect of TTO and its individual components was tested on confluent BGM cell monolayers using the MTS and sulforhodamine B (SRB) assays. Treatments were carried out for 2 h at 37 °C with concentrations of the different compounds ranging from 0.05% to 0.5%. The metabolic activity of treated cells was estimated using the MTS assay. A similar reduction of 50% cell viability (IC_50_) was observed at a concentration of 0.15% ± 0.02 for TTO and terpinen-4-ol and 0.28% ± 0.06 for γ-terpinene. In contrast, 1,8-cineole proved to have a small effect on cell viability, which could be detected using the MTS assay only at the higher concentration used (Figure 2). The SRB assay was used for cell proliferation determination, by measuring the cellular protein content, obtaining, as observed by the MTS assay, a similar concentration of growth inhibition GI_50_ for TTO and terpinen-4-ol and a slightly higher concentration for γ-terpinene (Figure 2). As observed from the graphs in Figure 2, the terpinen-4-ol and γ-terpinene compounds negatively affect cell viability at concentrations above 0.5% *v/v*. The adverse action of TTO is fully superimposable to that of terpinen-4-ol.

#### 2.1.4. Virucidal Activity

TTO activity was evaluated against the non-enveloped Coxsackievirus B4 (CVB4). The treatment of CVB4 viral suspension with TTO (5% *v/v*) was shown to have virucidal activity after 1 h of contact time with a percentage of viral reduction, in relation to viral load (TCID_50_/mL), ranging from 82.5% at 10^6^ TCID_50_/mL to 91.4% at 10^5^ TCID_50_/mL (Table 3). Three TTO components were tested at 10^6^ TCID_50_/mL. Terpinen-4-ol, with two log of viral reduction (% viral reduction of 99.1%), was more active than whole TTO whereas 1,8-cineole and γ-terpinene appeared to be less active in vitro than the whole oil (Table 3). However, the evaluated differences between the oil and its three components may not be clinically significant. According to cell viability data (Figure 2), due to the cytotoxicity of TTO and its tested components, the detection limit did not allow a higher viral reduction to be observed. No statistically significant difference was found between the vehicle (EtOH) and the mock-treated control. The observed virucidal activity, as previously demonstrated on human and animal coronaviruses [6], can be attributed to a direct effect on the extracellular viral particle, before penetration into a host cell. Proof of the virucidal activity of TTO opens the possibility of its use as a disinfectant (TTO 10%) and handwash formulation (TTO 5%) in hospital environments as well as a potentially useful topical treatment for cutaneous viral infections such as cold sores [6,7,36,47].

### 2.2. Simulations Results

#### 2.2.1. Interaction of TTO Components with a Model Bacterial/Fungal Membrane

As a model for TTO interaction with a bacterial surface, a system composed of the peptidoglycan glycosyltransferase protein (PDB ID: 3VMR) [48] embedded in a bacterial membrane (47% dipalmitoylphosphatidylglycerol, 40% cardiolipin, 8% stigmasterol and 5% ergosterol) was built using the CHARMM-GUI interface [49] (Figure S1). Due to its structural similarity to ergosterol, a key component of fungal cell membranes, the stigmasterol present in this model can provide valuable insights into how TTO interacts with fungal membranes. Therefore, the results obtained from this simulation can also be useful in understanding the influence of TTO on fungal membranes.

##### TTO Molecules Entry into the Membrane

**Figure 3:**
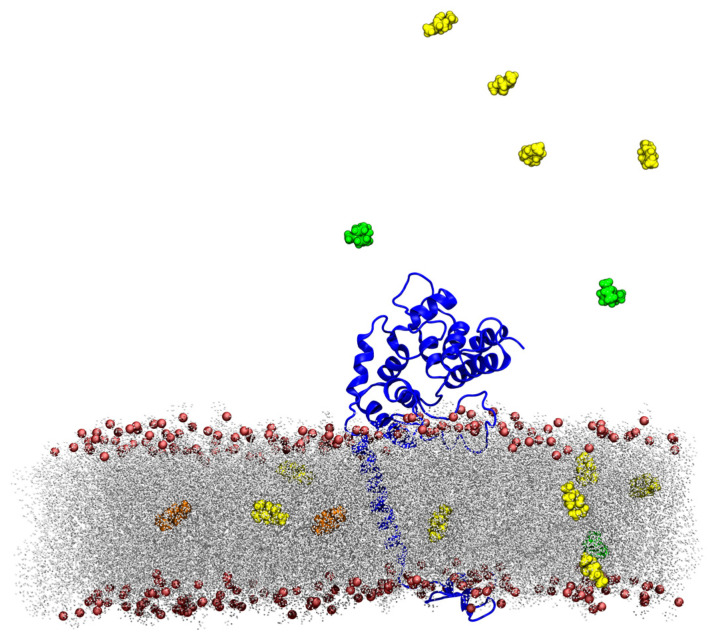
Snapshot extracted from the bacterial membrane–TTO simulation. The peptidoglycan glycosyltransferase is represented as a blue cartoon, while the membrane hydrophobic tails are shown as grey lines and the lipid polar heads as red spheres. TTO compounds are shown as spheres with γ-terpinene in orange, 1,8-cineole in green and terpinen-4-ol in yellow.

To verify if one or more TTO compounds penetrated the simulated lipid bilayer, we carried out two different analyses. From visual inspection, it was possible to observe that some TTO molecules entered the membrane (Figure 3) in the early stage of the simulation (first 30 ns of trajectory), reaching the interface between the two layers, where they moved only horizontally during the remaining 120 ns. No particular difference was detected in the ratio of terpinen-4-ol, γ-terpinene or 1,8-cineole molecules interacting with the membrane. In addition, a contact analysis was carried out to investigate whether TTO compounds preferred some specific lipid components of the membrane. For this purpose, an in-house script was created to analyze all the contacts established during the simulation between the system components. Table S1 reports the contacts with a high percentage of existence for the three TTO components with the lipid membrane. The results indicate that these compounds do not show any preference for the different lipid classes, and, moreover, none of these molecules directly interacts with ergosterol, allowing the hypothesis that the mechanism of action of TTO does not involve the destabilization of lipid rafts, as previously indicated [32].

##### Membrane Thickness Analysis

**Figure 4:**
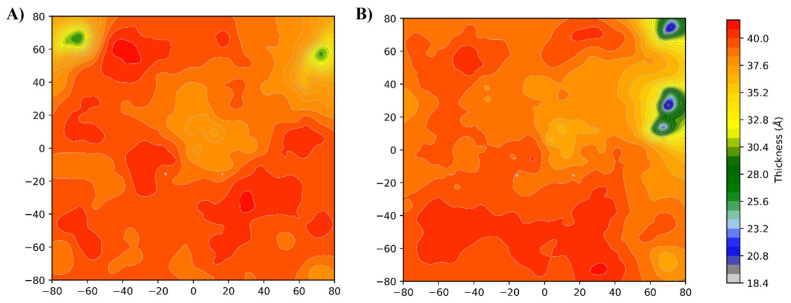
Heatmaps representing the thickness maps of the systems simulated in the absence (**A**) and presence (**B**) of TTO compounds.

A widely accepted hypothesis of the mechanism of action of TTO on membranes is the possibility that these compounds may alter the physical properties of the lipid bilayer [26,50,51]. The VMD Membrane Plugin [52] allows to generate a thickness/density profile, averaged over the simulation time, for all types of lipid membranes. Two heatmaps were generated for the simulated systems using the membrane thickness tool, describing the thickness profile along the membrane z-axis (Figure 4). The system simulated in presence of TTO (Figure 4B) showed an altered thickness profile characterized by the occurrence of thinner sections along the membrane, comparing with the membrane simulated in the absence of these molecules (Figure 4A). Moreover, membrane density profiles show that the presence of the TTO compounds transforms the density profile (red line) along the z-axis when compared with the reference system (black line) (Figure S2). This shift is directly correlated to an increase in local curvature of the membrane induced by the contact with the TTO molecules [53]. These data clearly indicate that the inclusion of the TTO compounds alters the membrane behavior, suggesting that the function of the embedded proteins may be affected by the altered membrane properties, as also reported in the literature [26,27]. To validate this hypothesis, a series of structural analyses were performed on the simulated peptidoglycan glycosyltransferase enzyme, to quantify the consequences of the variations observed at the membrane level.

##### Peptidoglycan Glycosyltransferase RMSD and RMSF Analyses

Figure S3 shows the RMSD values, calculated for all the protein atoms as a function of time, for the reference and TTO systems (black and red lines, respectively). The results indicate that the protein simulated in the presence of TTO compounds reaches higher RMSD values, suggesting the occurrence of conformations deviating from those sampled in the reference system. This difference in stability could represent a direct consequence of the membrane alterations generated by the TTO compounds, which may cause a decrease, or even a deactivation of the protein function, providing a further interpretation supporting the hypothesized mechanism of action of TTO. Figure S4 shows the per-residue RMSF values calculated for the protein in the reference and TTO systems (black and red lines, respectively). As shown, the N-terminal region of the protein and the residues in the range 100–150 display a different fluctuation profile. While the flexibility of the N-terminal segment is neglectable, being frequently observed for terminal residues, that observed for residues 100–150 can be directly related to a potential inactivation of the enzyme, since this region includes the active site of the peptidoglycan glycosyltransferase, composed by Lys140, Arg148 and Glu100 (black filled circles in Figure S4). Alterations in the active site due to membrane incorporation of TTO compounds may inactivate this enzyme, which is essential for peptidoglycan and bacterial cell wall biosynthesis, inducing the reduction of bacterial growth.

##### PCA Analysis

**Figure 5:**
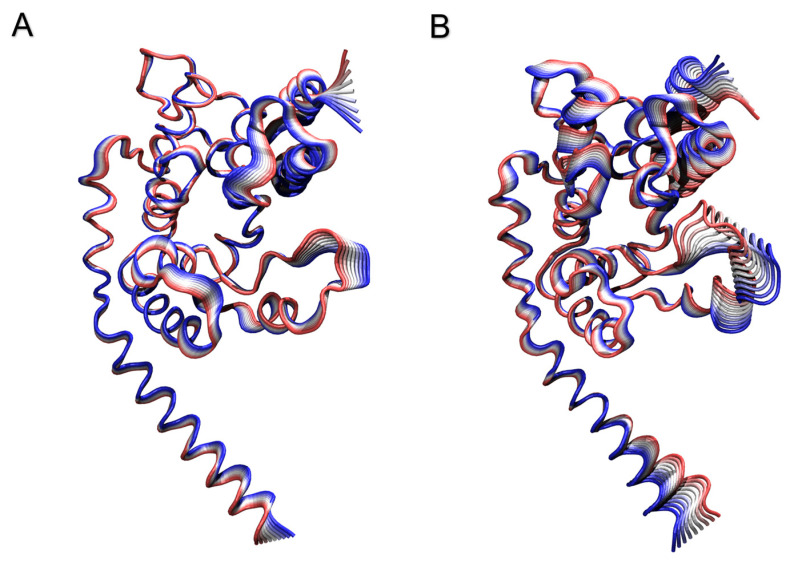
Projections of the motion identified along the first eigenvector over the 3D structure of the protein simulated in the absence (**A**) and presence (**B**) of the TTO compounds. The direction of motion is described by the color ranging from red to blue, while the amplitude of motion by the width of the ribbon.

The covariance matrices, generated for both simulated systems through PCA analysis, were converted using a Python script into the corresponding dynamic cross-correlation maps (Figure S5A,B). Positive values imply a positive correlation (i.e., residues move in the same direction), while negative ones represent a negative correlation (i.e., residues move in the opposite directions). As shown in the maps, the presence of the TTO compounds induces a change in the cross-correlation patterns between the protein residues (Figure S5B). This results in a loss of both positive and negative correlations for the entire protein, suggesting that, in the two systems, the enzyme samples a significantly different conformational space. Confirming this observation, the 2D projections of the motions along the first two eigenvectors for both systems indicate that the protein samples a non-overlapping conformational space when simulated in the absence or presence of the TTO compounds (Figure S6). Moreover, projection of the motions described by the first eigenvector on the 3D structure (Figure 5) confirms that the protein in the TTO system is characterized by motions that are different both in amplitude and direction, and which can be linked to an altered enzyme functionality.

##### Salt Bridges and Hydrogen Bonds

Variation of protein’s internal motions is correlated with a deep change in its electrostatic networks. In detail, salt bridges and hydrogen bonds persistence were analyzed for both systems over the entire simulation time. Table S2 shows the percentage of persistence of the salt bridges identified in the two systems. In the case of the TTO system, some salt bridges are lost in the protein (i.e., Asp105-Lys107, Asp121-Arg120, Asp139-Lys134, Asp141-Arg142, Glu241-Arg120), with the establishment of two novel salt bridges (Asp178-Lys216, Asp62-Lys149), not detected in the reference system. A different pattern can also be observed for the hydrogen bonds networks (Table S3), with around 20 hydrogen bonds being lost in the TTO system. This confirms a structural/dynamical and, therefore, a functional alteration of the protein inserted into the lipid bilayer in the presence of TTO compounds. 

#### 2.2.2. TTO Compounds Interactions with the Coxsackievirus B4 Viral Capsid

**Figure 6:**
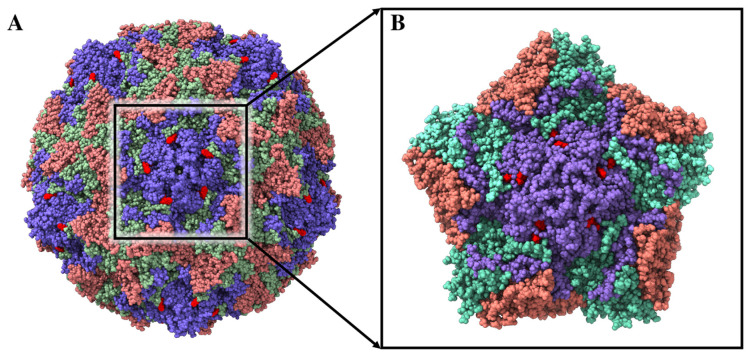
(**A**) The structure of Coxsackievirus B4 in complex with capsid binder CP17 (red spheres). VP1, VP2 and VP3 are represented by violet, red and salmon spheres, while VP4 is not shown being below the plane formed by the other three structures. (**B**) Complex between terpinen-4-ol (red spheres) and the five interacting protomers.

The interaction of TTO molecules within the CP17 binding site, located at the interface of the protomer structures forming the capsid of Coxsackievirus B4 (PDB ID: 6ZCL and 6ZMS) [54] (Figure 6), has been evaluated using molecular docking simulations. The docking results indicate that two TTO molecules can simultaneously bind within the inter-protomer binding site with similar energies (Table 4), with a small preference for terpinen-4-ol. Given the complexity of the system and the subsequent long simulation times, we restricted the analysis by performing a 150 ns molecular dynamics simulation of a virus portion including only five viral protomers in complex with terpinen-4-ol molecules, showing the best interaction energies in the molecular docking simulations (Figure 6B). Since these capsid binders are small molecules that bind tightly and specifically to conserved capsid features to interfere with virus entry or uncoating, we focused our analyses on the stability of terpinen-4-ol molecules within the binding sites, calculating their interaction energy for the five identical pockets of our reduced system. 

Notably, three out of five complexes were characterized by the loss of the terpinen-4-ol molecule directed outwards of the pocket, which started to interact randomly over the protomer surfaces, while the other two were stable until the end of the simulation. However, the results of MM/GBSA calculations [55] performed over the last 50 ns of simulation time, confirmed a strong interaction between the terpinen-4-ol and the interprotomer binding pockets, which are characterized by an average free energy of interaction of −11.7 and −27.0 kcal/mol for the single and double terpinen-4-ol binding modes inside the pocket, respectively (Table 5). As expected for terpinen-4-ol, the main contribution to the interaction energy comes from the VdW term, which is a rough estimate of the hydrophobic interactions. These results suggest that the molecules present in the TTO may behave as capsid binders, occupying the interprotomer sites and stabilizing the viral capsid. In this way, TTO molecules could interfere with the virus entry phase by hindering the viral particle from undergoing the conformational changes required to uncoat its genome and progress with the infection [54].

## 3. Discussion

The therapeutic properties of *M. alternifolia* have been known for years [56]. This work aimed to speculate about its molecular mechanism of action and clarify which are the alterations, at the atomistic level, that TTO molecules can induce in viral capsids or in bacterial membranes and proteins. The results obtained here allowed us to propose a model underlying the antimicrobial activity of the components of the oil extracted from the *M. alternifolia* plant. Regarding the bacteria, both *S. typhimurium* and *S. aureus* are sensitive to TTO compounds, even if at different dilutions and with different abilities to recover their growth (Figure 1). γ-terpinene was found to be not effective at all in both species, while terpinen-4-ol has the highest growth inhibitory effect. The effect of 1,8-cineole appears to be quite different on the two species: *S. typhimurium* growth is almost completely inhibited, while *S. aureus* is still able to grow at low concentration, even after a twelve-hour delay (Figure 1). The TTO components, tested individually, could also effectively inhibit fungal growth, although their antifungal efficacy was slightly lower (one-fold) than that of the total plant extract (Table 2). In a non-enveloped virus such as the Coxsackievirus B4, the virucidal activity was demonstrated for the whole oil and its component terpinen-4-ol, while the other two components were not very effective (Table 3).

Our simulations suggest that the TTO molecules may act as capsid binders, occupying the interprotomer sites (Figure 6), stabilizing the viral capsid and hindering the virus particle from undergoing the conformational changes required to uncoat the genome and progress with the infection [54]. Moreover, given their small size, relatively large hydrophobicity and low molecular weight, these compounds can easily penetrate bacterial or fungal lipid bilayers (Figure 3). A contact analysis has been carried out for each TTO molecule to assess the presence of any preferential affinity of these molecules towards bacterial/fungal membrane components (Table S1), allowing the exclusion of any preferential contact with ergosterol and stigmasterol. Once entered, the TTO compounds cause significant and non-specific changes in the membrane, decreasing its thickness (Figure 4) and inducing a shift in the density profile (Figure S2). These results indicate that the presence of TTO can alter the chemical/physical characteristics of the membrane and suggest that the embedded proteins may be affected by these changes. As proof of this hypothesis, several analyses were carried out on the structural/dynamical properties of the *S. aureus* peptidoglycan glycosyltransferase, embedded in the membrane. The RMSD analysis (Figure S3) highlights a lower stability of the enzyme in the TTO system, while the RMSF analysis (Figure S4) indicates that residues fluctuation profiles and, thus, protein flexibility in the region including residues 100–150, are affected by the presence of TTO molecules. This different flexibility may be directly linked to a possible inactivation of the enzyme. In fact, the active site of the peptidoglycan glycosyltransferase consists of the residues Lys140, Arg148 and Glu100, which are affected by the changes in the surrounding environment. The PCA analysis carried out on peptidoglycan glycosyltransferase showed how the TTO compounds are able to induce a change in the protein cross-correlation pattern, with a loss of both positive and negative correlations (Figure S5). These data suggest that in the two simulations the protein samples two different conformational spaces, losing internal motions that are essential for its functionality (Figures S5 and S6). By projecting the motions on the protein 3D structure (Figure 5), we also observed that in the case of the TTO system most of protein motions have different direction and amplitude when compared with the reference system, further confirming the direct influence exerted by membrane deformations induced by the presence of TTO compounds. It can be therefore hypothesized that the variation of protein internal motions may also be correlated with an alteration of its electrostatic interaction networks. Salt bridge (Table S2) and hydrogen bond (Table S3) analyses, in fact, confirmed that the electrostatic networks are significantly altered in the presence of TTO compounds. Similar effects have been observed for the SARS-CoV-2 spike glycoprotein and viral membrane when simulated in the presence of these TTO compounds [6].

## 4. Materials and Methods

### 4.1. Experimental Methods

#### 4.1.1. Antimicrobial Agents’ Preparation

TTO, 1,8-cineole, γ-terpinene and terpinen-4-ol were purchased from Sigma-Aldrich (St. Louis, MO, USA) and had a purity of 98%. Fluconazole powder (Sigma-Aldrich) was prepared in sterile saline at a concentration of 1.0 mg/mL and frozen at −80 °C.

#### 4.1.2. Cell Cultures and Viability Assays

BGM cells, derived from African green monkey kidney, were grown in DMEM (Dulbecco’s modified Eagle’s medium) (Biowest, Miami, FL, USA), supplemented with 10% FBS (Gibco, Paisleg, UK), 1 mM L-glutamine (Sigma-Aldrich, St Louis, MO, USA), 1 mM sodium pyruvate (Biowest, Miami, FL, USA) and 100 U/mL penicillin–streptomycin (Euroclone, Devon, UK). BGM cells were seeded in 96-well plates at 2 × 10^4^ cells per well, grown to confluence and treated with different concentrations of TTO, terpinen-4-ol, **γ**-terpinene and 1,8-cineole for 2 h at 37 °C. Oil compounds were dissolved in 80% ethanol at an oil:ethanol ratio of 1:2 for TTO, terpinen-4-ol and 1,8-cineole, and 100% ethanol for **γ**-terpinene at the same oil:ethanol ratio and serial dilutions were made in DMEM 10% FBS. Cell viability was evaluated using the Sulforhodamine B (SRB) assay and the 3-(4,5-dimethylthiazol-2-yl)-5-(3-carboxymethoxyphenyl)-2-(4-sulfophenyl)-2H-tetrazolium (MTS) assay (Promega, Madison, WI, USA). Briefly, for SRB assay, cells were stained with 0.04% SRB solution in 1% acetic acid for 10 min at room temperature, as described [57]. The protein-bound dye was dissolved in 10 mM Tris base solution, and absorbances at 510 nm were recorded. The MTS assay was performed following the manufacturer’s instruction and the absorbance was measured at 492 nm. For both assays, absorbance was measured using a Multiskan Ascent 96/384 Plate Reader (MTX Lab Systems, Vienna, VA, USA). Control cells were incubated in the presence of the same concentration of ethanol used for oil dilutions. Experiments were performed in triplicates, and values were normalized to control condition. Statistical analysis was performed with ANOVA test using GraphPad Prism 6.

#### 4.1.3. Bacterial Strains and Media

Laboratory collections of *Salmonella enterica* serovar Typhimurium (ATCC14028) and *Staphylococcus aureus* (SH1000) were employed for growth assays. Both strains were grown in Luria Bertani (LB) medium (Bacto tryptone 10 gL^−1^, Yeast extract 5 gL^−1^, NaCl 10 gL^−1^), solidified by the addition of 1.5% (*w/v*) agar when needed. All media reagents were purchased from BD (Franklin Lakes, NJ, USA).

#### 4.1.4. Growth Curves Assays and MIC Determination

Single colonies, grown on LB agar plates from glycerol stock samples, were inoculated in 2 mL LB and incubated for 5 h at 37 °C with aeration. Inocula were then diluted 1:1000 in fresh LB medium plain or supplemented with increasing amounts of TTO, terpinen-4-ol, γ-terpinene and 1,8-cineole. Triplicates of each sample were plated on a 96-well microtiter plate, and bacterial growth was monitored in a Sunrise microplates reader (Tecan, Männedorf, Switzerland) at 37 °C with periodic agitation, by recording Optical Densities at 595 nm (OD595) every hour for at least 18 h. For MIC determination, the OD595 after 18 h of growth of treated samples compared to untreated were considered.

#### 4.1.5. *Candida* Strain and Growth Conditions

*C. albicans* laboratory reference strain ATCC 2091 was grown on Sabouraud dextrose agar (Difco Laboratories, Detroit, MI, USA) supplemented with chloramphenicol for 24 h at 30 °C. After incubation, *Candida* yeasts were harvested by washing the slant culture with sterile saline, and the cell density of the fungal suspension was estimated by direct cell count, using a Burker chamber, and adjusted to a cell density of 1 × 10^5^/mL.

#### 4.1.6. Determination of Minimum Inhibitory and Fungicidal Concentration

The antifungal activity of TTO and its related components against *C. albicans* was assessed using the broth microdilution method, as indicated by the Clinical and Laboratory Standards Institute (CLSI) [46], with some modifications. Briefly, RPMI 1640 with 2% glucose and L-glutamine (Sigma-Aldrich, St. Louis, MO, USA) buffered to pH 7.0 and added with tween 20 (0.5% *v*/*v*) was used as fungal culture medium. Tween 20 was included to improve oil solubility [22]. At this concentration, no inhibitory effect was obtained on *C. albicans* growth by the detergent. A 100 μL volume of *C. albicans* suspension was added to 96-well flat-bottom plates. Two-fold serial dilutions of TTO and its components, including 1,8-cineole, γ-terpinene and terpinen-4-ol, were prepared to final concentrations ranging from 4% *v/v* to 0.0075% *v/v*. Aliquots of 100 μL of the compounds were dispensed into each well. Fluconazole, ranging from 256 to 0.03 µg/mL was chosen as the control in the study. Positive (100 μL of culture medium plus 100 μL of *C. albicans*) and negative controls (200 μL of culture medium alone or 100 μL of culture medium plus 100 μL of each compound in the absence of *C. albicans*) were included in all experiments. The plates were incubated with agitation at 30 °C for 24 h. Minimum inhibitory and fungicidal concentrations (MIC and MFC, respectively) were determined in three independent experiments carried out in triplicate. The MIC_50_ was determined spectrophotometrically at 510 nm with an enzyme-linked immunosorbent assay (ELISA) reader and was defined as the lowest concentration of the compounds that inhibited 50% of fungal growth compared with the drug-free control. The MFC was defined as the lowest concentration resulting in the death of 99.9% or more of the initial inoculum. To determine MFCs, 50 μL of broth was taken from the wells without microbial growth, inoculated onto Sabouraud’s dextrose agar (SDA) and incubated at 37 °C. After 24 h, the colony forming unit (CFU) were counted to assess viability.

#### 4.1.7. Cells and Virus

Buffalo Green Monkey cells (BGM) were grown in Eagle minimum essential medium (MEM) supplemented with 10% inactivated newborn calf serum (Euroclone, Devon, UK), 100 U/mL penicillin (Pharmacia, Sweden), 100 μg/mL streptomycin, 2 mM L-glutamine and 1% sodium bicarbonate. The Coxsackievirus B4 is routinely cultivated in our laboratory on BGM cells. Monolayers grown in 25 cm^2^ flask were infected with 0.1 MOI of the virus, and after 1 h adsorption at 37 °C, 6–8 mL of fresh medium was edited. When the cells showed a clear cytopathic effect, the flask was freeze-thawed three times, the supernatant was centrifuged at 3000 rpm/15 min/room temperature, and the supernatant titered and frozen at −80 °C till use. For CVB4 infectivity assay, BGM cells grown on 96-well plates (2 × 10^4^ cells/well) were incubated with serial dilutions of viral suspension for 48 h at 37 °C in 5% CO_2_, and TCID_50_ titer (50% tissue culture infective dose) was determined according to the Reed and Muench method [58].

#### 4.1.8. Cell Culture and Treatments

BGM cells were plated onto 96-well plates at density of 2 × 10^4^ cells. Each compound was dissolved in ethanol 80%. In detail, 1 vol. of each oil was dissolved in 2 vol. of ethanol 80%, reaching the concentrations of 33.3% and 53.3%, respectively. The following dilutions were performed in MEM 2% FCS. Each condition was tested in duplicate.

#### 4.1.9. Pre-Treatment of Viral Suspension

To test the virucidal activity of TTO, 1 × 10^6^ or 1 × 10^5^ TCID_50_/_mL_ of CVB4 were mixed to a final concentration of 5% TTO in MEM 2% FCS in 100 μL of total volume. The dilution started from 33.3% in 53.3% EtOH till 5% TTO in 8% EtOH. The individual TTO components were tested with 1 × 10^6^ TCID_50_/_mL_ of viral suspension as described for whole oil with starting dilution as indicated in cell viability assays section. The contact time between CVB4 and the whole TTO or its single components was 60 min. After 1 h of contact time, the virucidal activity was blocked by the addition of 900 μL of MEM 2% FCS, and the titrations carried out using TCID_50_ infectivity assay. Briefly, the endpoint dilution titration was performed by incubating ten-fold serial dilutions of the CVB4–TTO mixture, starting from a non-cytotoxic concentration, on BGM cell monolayers in 96-well culture plates at 37 °C in 5% CO_2_. The single TTO components were titrated using the same procedure as the CVB4–TTO mixture. Vehicle (EtOH) and control virus mock-treated (MEM 2% FCS) were included in each test virucidal activity assay. At 48 h, post-infection (p.i.) viral titers were determined using the Reed and Muench method and expressed as a percentage of viral inhibition from an average of three experiments.

### 4.2. Computational Methods

#### 4.2.1. Systems Preparation

The peptidoglycan glycosyltransferase (or murein synthase), a small protein of 261 amino acids (PDB ID: 3VMR) [48] was selected as a model membrane protein and inserted in a fragment of bacterial membrane, composed by 47% of dipalmitoylphosphatidylglycerol (DPPG), 40% of cardiolipin (TMCL1), 8% of stigmasterol and 5% of ergosterol (Figure S1). The Membrane Builder tool of CHARMM-GUI [49] was used to build the membrane–protein system, optimizing protein orientation within the bilayer through the OPM server. As the stigmasterol molecule present in our model membrane system only has small structural differences compared to the ergosterol molecule, the results obtained are also directly transposable to the cytoplasmic membranes of fungi. In fact, this small difference would not justify a different behavior of the TTO compounds towards the fungal membrane. Structures of three most representative TTO compounds (terpinen-4-ol, 1,8-cineol, γ-terpinene) have been retrieved from the PubChem database [59] (https://pubchem.ncbi.nlm.nih.gov/, accessed on 30 November 2022) (Figure S1). The structure of an asymmetric unit of the Coxsackievirus B4 (PDB ID: 6ZMS) [45], selected as a model virus, was superimposed with the Coxsackievirus B3 viral capsid in complex with capsid binder CP17 (PDB ID: 6ZCL) [54], to obtain a final structure composed by 60 repeating asymmetric units each consisting of the four structural proteins, VP1, VP2, VP3 and VP4 (Figure 6A). The asymmetric unit was used as a receptor for molecular docking experiments to check for the possible interactions of TTO molecules within the same binding site of the capsid binders. To reduce the complexity of the system, the MD simulation of the TTO–capsid complex was restricted to five interacting protomers for a total of 20 structural proteins (Figure 6B).

#### 4.2.2. Peptidoglycan Glycosyltransferase Simulations

Two systems were simulated, including the peptidoglycan glycosyltransferase inserted in the membrane in the absence or presence of several molecules of the three main TTO compounds (Table S4). TTO molecules were randomly inserted around the protein–membrane system through the UCSF Chimera 1.17.1 program [60]. Topology and coordinate files were generated through the VMD software [61], parametrizing the membrane and the protein using the CHARMM36 forcefield [62,63,64] and the TTO compounds using the CGenFF web-server (https://cgenff.umaryland.edu, accessed on 30 November 2022) and the CHARMM general force field [64]. Structures were inserted in a triclinic box, solvated with TIP3P water molecules [65], imposing a distance from the box sides between 20.0 and 60.0 Å for the system with the TTO molecules and between 20.0 and 40.0 Å for the system in the absence of TTO. Systems were then neutralized adding NaCl ions at a concentration of 0.15 mol/L. To stabilize the complex, eight minimization stages, each including 2000 steps, were performed. An initial constraint of 5.0 kcal/mol was applied on each atom, then halved during subsequent stages, and finally removed in the last one. The minimized structures were thermalized using the canonical ensemble NVT and a timestep of 1.0 fs, starting from 0 K and gradually increasing temperature by 10 K every 30 ps, until the final temperature of 300 K was reached. Production phase was then carried out in an NPT ensemble, increasing the timestep to 2.0 fs. Electrostatic interactions were calculated every 4.0 fs through the Particle-Mesh Ewald method [66], using a grid spacing of 1.0 Å. The cut-off for non-bonded interactions was set to 12.0 Å, updating the neighbors’ list every 20 steps, with a distance of 14.5 Å for the atoms’ inclusion. Temperature was kept constant at 300 K using the Langevin thermostat [67] with a coupling coefficient of 5.0 ps applied to all atoms. For the pressure control, the Langevin piston Nosè–Hoover method was used [68,69], setting the pressure to 1.0 atm. Systems were simulated for 150 ns using the NAMD 2.13 program [70] on the ENEA CRESCO6 HPC cluster [71], saving the coordinates every 2500 steps.

#### 4.2.3. Molecular Docking Simulations

The structure of the five interacting protomers (five copies of VP1, VP2, VP3 and VP4 proteins) was used as a receptor of the molecular docking experiments. The CP17 molecule, a benzenesulfonomide derivative, that potently inhibits the CVB3 was present in the cryo-EM structure [54] and was removed from the complex. The structures of the TTO molecules downloaded from the PubChem database were converted in 3D using the OpenBabel routines [72]. Receptor and drug structure files were converted into pdbqt format using the prepare_receptor4.py and prepare_ligand4.py tools of the AutoDockTools4 program [73]. Protein–ligand molecular docking simulations were performed using the AutoDock Vina 1.2.3 program [74], considering the possibility that multiple molecules could bind within the defined binding site. A molecular docking simulation, including ten docking runs, was performed using a box of size x = 20.25 Å; y = 22.15 Å; z = 21.35 Å, centered over the CP17 binding site for each TTO molecule combination. To increase the accuracy of binding pose estimation, 10 receptor residue side chains around the binding site were considered flexible.

#### 4.2.4. Coxsackievirus B4 Simulation

As a test bed, the complex between the five protomers and terpinen-4-ol molecules was simulated for 150 ns using classical MD simulation. Terpinen-4-ol molecules were firstly docked in one of the binding sites identified over the capsid protomer surface [54], and the final complex was replicated for the other four protomers using the UCSF Chimera 1.17.1 program [60]. Topology and coordinate files were generated through the VMD 1.9.3 software, parametrizing the protein using the CHARMM36 force field and the TTO compounds using the CGenFF web-server and the CHARMM general force field as described for the peptidoglycan glycosyltransferase. Structures were inserted in a triclinic box, solvated with TIP3P water molecules, imposing a distance from the box sides of 20 Å. Systems were then neutralized adding NaCl ions at a concentration of 0.15 mol/L. The equilibration and production MD phase was performed as described for the other systems.

#### 4.2.5. Trajectories Analyses

Root mean square deviation (RMSD), root mean square fluctuations (RMSF) and principal component analysis (PCA) were carried out using the GROMACS 2021 MD package [75]. The principal component analysis (PCA), or essential dynamics [76], was performed on Cα atoms of the peptidoglycan glycosyltransferase to highlight differences in the correlation patterns of the protein between the two simulations. The analysis is based on the diagonalization of the covariance matrix, built from the atomic fluctuations after the removal of the translational and rotational movement, allowing the identification of the main 3N directions along which most of the protein motion is defined [76]. The covariance matrix was calculated using the GROMACS 2021 *covar* module, and the first two generated eigenvectors were analyzed using the *anaeig* module. The projections of the motions were calculated along the first eigenvector. Salt bridges and hydrogen bonds in the peptidoglycan glycosyltransferase enzyme were identified through the VMD 1.9.3 [61] SaltBridge and Hbonds plugins, respectively. Salt bridges were considered up to a distance of 8.0 Å with a percentage of persistence varying at least 10% between the two systems, and were processed through an in-house program in C. The Membrane Analysis tool [52], implemented in the VMD software, was used to calculate membrane thickness based on the mass distributions of the lipids phosphorus atoms. Molecular mechanics/generalized Born and surface area continuum solvation (MM/GBSA) analyses [55] were performed using the MMPBSA.py.MPI program implemented in the AMBER16 software [77] on three nodes of the ENEA HPC cluster CRESCO6 [71], setting the ionic strength to 0.15 M. Images were created using the VMD 1.9.3 [61] or Chimera 1.17.1 [60] software.

## 5. Conclusions

In this work, experimental and in silico tests on various microorganisms were performed to highlight the remarkable antimicrobial effect of TTO, providing atomistic mechanisms to elucidate the macroscopically observed activity. Based on the obtained data, we suggest that the ability of the TTO to interfere with microbial growth and viral infection could be due to a fast and non-specific interaction with the components present on the outer surface of the evaluated species, even if it is not possible to exclude the presence of specific interaction with other macromolecules. Undoubtedly, these simulation methods are characterized by intrinsic limitations since they can describe only a small portion of the system and, given the high computational cost of calculations, only include some of the molecular components of bacteria, fungi and viruses. On the other hand, MD sampling strategies and force fields, in concert with complementary experimental methods, currently represents a valuable tool for characterizing biomolecular interactions and processes. In conclusion, these findings support the possibility of developing topical antimicrobial treatments based on TTO due to its broad-spectrum activity, chemical heterogeneity and its efficacy in interfering with key microbial structures. In this regard, it should also be emphasized that the mechanism of action of TTO makes it unlikely for pathogens to develop resistance mechanisms against TTO.

## Figures and Tables

**Table 1:** MIC_100_ values of TTO and its components against *S. typhimurium* and *S. aureus*. MIC_100_ (minimal inhibitory concentrations preventing visible growth after 18 h) are expressed in % *v*/*v*.

Treatment (18 h)	*S. typhimurium* (MIC_100_)	*S. aureus* (MIC_100_)
TTO	0.3	0.8
1,8-cineole	0.8	>1.6
γ-terpinene	>0.8	>1.6
terpinene-4-ol	0.2	0.2

**Table 2:** MIC and MFC values of TTO and its components against *C. albicans* ATCC 2091. MICs = minimal inhibitory concentrations are expressed in % *v/v* (TTO, 1,8-cineole, γ-terpinene and terpinen-4-ol) or µg/mL (fluconazole); MFCs = minimum fungicidal concentrations.

Treatment (24 h)	MIC50	MFC
TTO	0.125	0.25
1,8-cineole	0.25	0.5
γ-terpinene	0.25	0.5
terpinene-4-ol	0.25	0.5
fluconazole	0.5	-

**Table 3:** Virucidal activity of TTO and its components against the non-enveloped Coxsackievirus B4 (CVB4), reported as percentage of viral reduction in relation to viral load (TCID_50_/mL).

	% Viral Reduction	
Sample	10^6^ TCID_50_/mL	10^5^ TCID_50_/mL
TTO (5% *v/v*)	82.5 ± 4.4	91.4 ± 6.6
terpinen-4-ol (5% *v/v*)	99.1 ± 3.1	N.D.
1,8-cineole (5% *v/v*)	20.0 ± 0.3	N.D.
γ-terpinene (5% *v/v*)	48.3 ± 4.7	N.D.

**Table 4:** Binding energy of TTO molecules within the interprotomer binding pocket on the capsid of Coxsackievirus B4.

Molecules	Binding Energy (kcal/mol)
terpinen-4-ol/terpinen-4-ol	−9.9
terpinen-4-ol/γ-terpinene	−9.7
terpinen-4-ol/1,8-cineole	−9.7
1,8-cineole/1,8-cineole	−8.9
γ-terpinene/γ-terpinene	−9.6
γ-terpinene/1,8-cineole	−8.8

**Table 5:** Results of the MM/GBSA analyses of the MD trajectories of the five protomers in complex with the terpinen-4-ol molecules.

Binding Site	VdW (kcal/mol)	Electrostatic (kcal/mol)	Interaction Energy (kcal/mol)
Site 1 (1 terpinen-4-ol)	−15.6	−1.9	−9.6
Site 2 (1 terpinen-4-ol)	−16.6	−1.9	−10.6
Site 3 (2 terpinen-4-ol)	−40.7	−4.9	−26.3
Site 4 (2 terpinen-4-ol)	−40.2	−5.5	−27.7
Site 5 (1 terpinen-4-ol)	−18.5	−2.9	−14.8

## Data Availability

Not applicable.

## Supplementary Materials

The following supporting information can be downloaded at: https://www.mdpi.com/article/10.3390/ijms241512432/s1.
