# Supplementary material for: Deciphering the Broad Antimicrobial Activity of Melaleuca alternifolia Tea Tree Oil by Combining Experimental and Computational Investigations
Source: Int J Mol Sci. 2023 Aug 4;24(15):12432. doi: 10.3390/ijms241512432 (PMC10420022; doi:10.3390/ijms241512432)
Supplement: Supplementary file 1 [file ijms-24-12432-s001.zip › ijms-2478714-supplementary.pdf]

SUPPLEMENTARY MATERIALS

Deciphering the Broad Antimicrobial Activity of *Melaleuca alternifolia* Tea Tree Oil by Combining Experimental and Computational Investigations

Federico Iacovelli [1,†], Alice Romeo [1,†], Patrizio Lattanzio [1], Serena Ammendola [1], Andrea Battistoni [1], Simone La Frazia [1], Giulia Vindigni [2], Valeria Unida [2], Silvia Biocca [2], Roberta Gaziano [3], Maurizio Divizia [4] and Mattia Falconi [1,*]

1→Department of Biology, University of Rome Tor Vergata, Via della Ricerca Scientifica 1, 00133 Rome, Italy; federico.iacovelli@uniroma2.it (F.I.); alice.romeo@uniroma2.it (A.R.); patriziolattanzioo@gmail.com (P.L.); serena.ammendola@uniroma2.it (S.A.); andrea.battistoni@uniroma2.it (A.B.); simone.la.frazia@uniroma2.it (S.L.F.)
2→Department of Systems Medicine, University of Rome Tor Vergata, Via Montpellier 1, 00133 Rome, Italy; giuliavindy@hotmail.it (G.V.); valeria.unida@gmail.com (V.U.); biocca@med.uniroma2.it (S.B.)
3→Microbiology Section, Department of Experimental Medicine, University of Rome Tor Vergata, Via Montpellier, 1–00133 Rome, Italy; roberta.gaziano@uniroma2.it
4→Department of Biomedicine and Prevention, University of Tor Vergata, 00133 Rome, Italy; divizia@uniroma2.it
*→Correspondence: falconi@uniroma2.it; Tel.: +39-06-7259-4025
†→These authors contributed equally to this work.

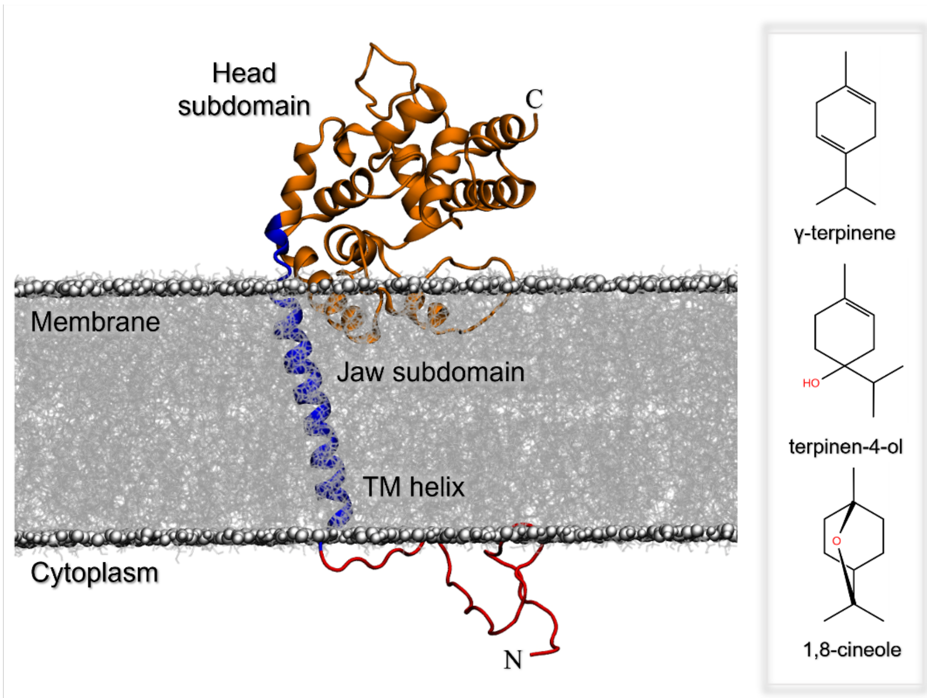

Figure S1. The peptidoglycan glycosyltransferase structure, represented as cartoon, inserted in a membrane bilayer. Lipid tails are shown as lines and polar heads as spheres. The 2D structures of the three main TTO compounds selected for the MD simulations are represented on the right.

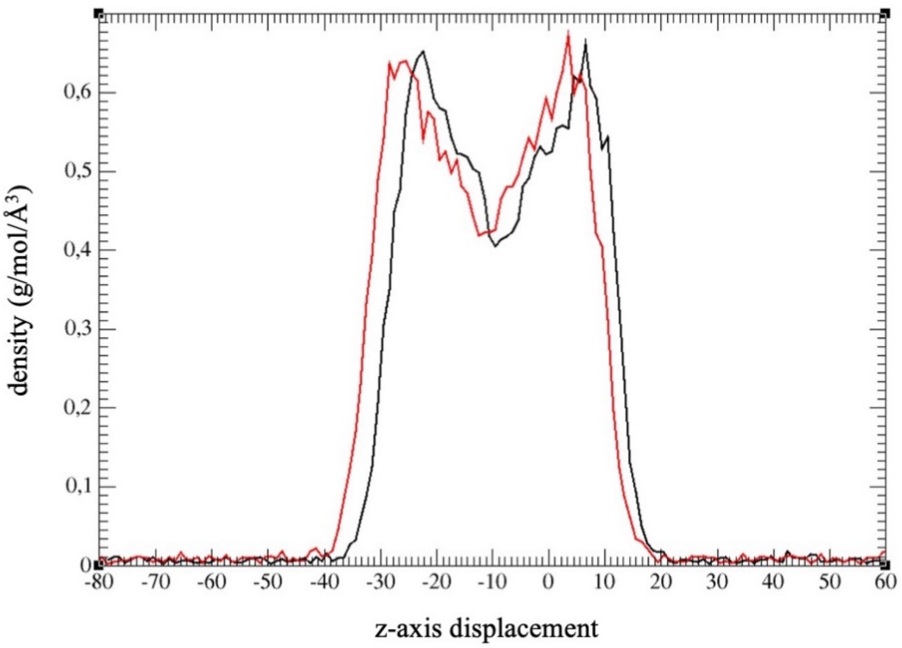

Figure S2. Density profile of the bacterial membrane calculated along the z-axis for the systems simulated in the absence (black lines) or presence (red lines) of TTO compounds.

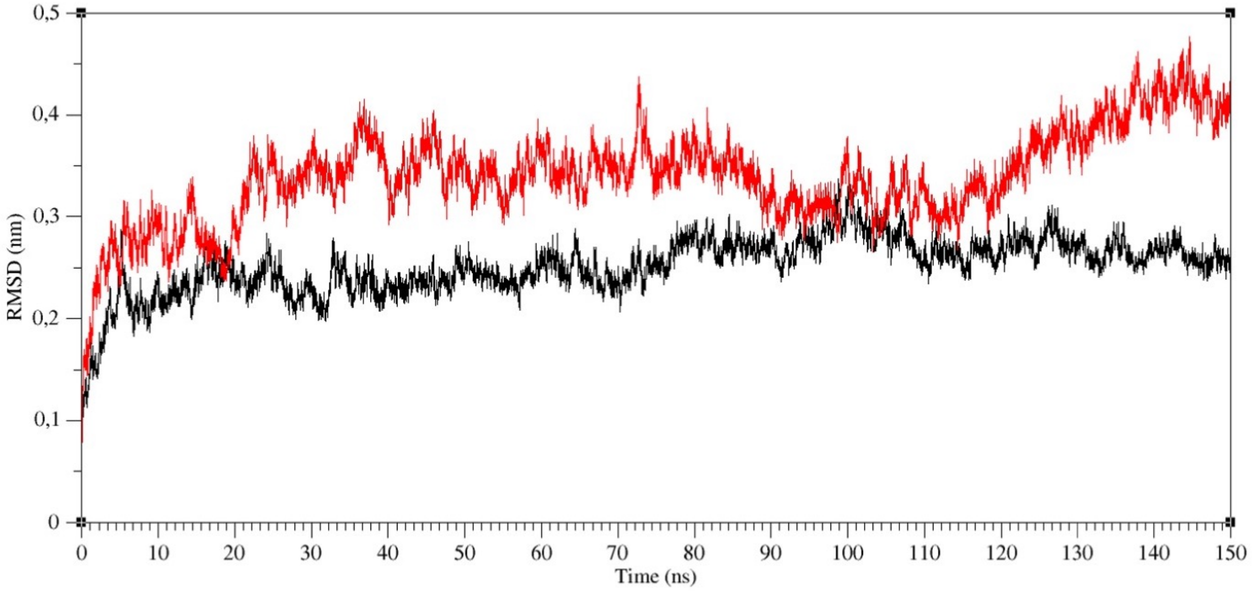

Figure S3. RMSD as a function of simulation time calculated for the peptidoglycan glycosyltransferase protein embedded in the lipid bilayer, in the absence (black line) or presence (red line) of TTO compounds.

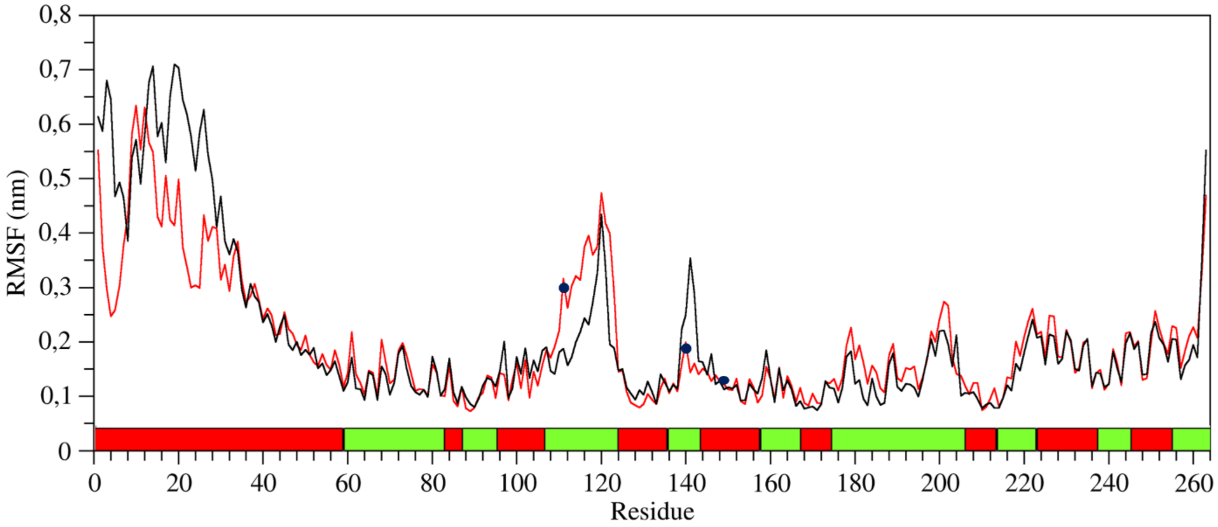

Figure S4. Per-residue RMSF analysis of the protein simulated in absence (black lines) or presence (red lines) of TTO molecules. The coloured bar above the x-axis indicates the secondary structure adopted by the protein, with the loop regions represented in red and the α-helices in green. The black filled circles indicate the residues making up the active site.

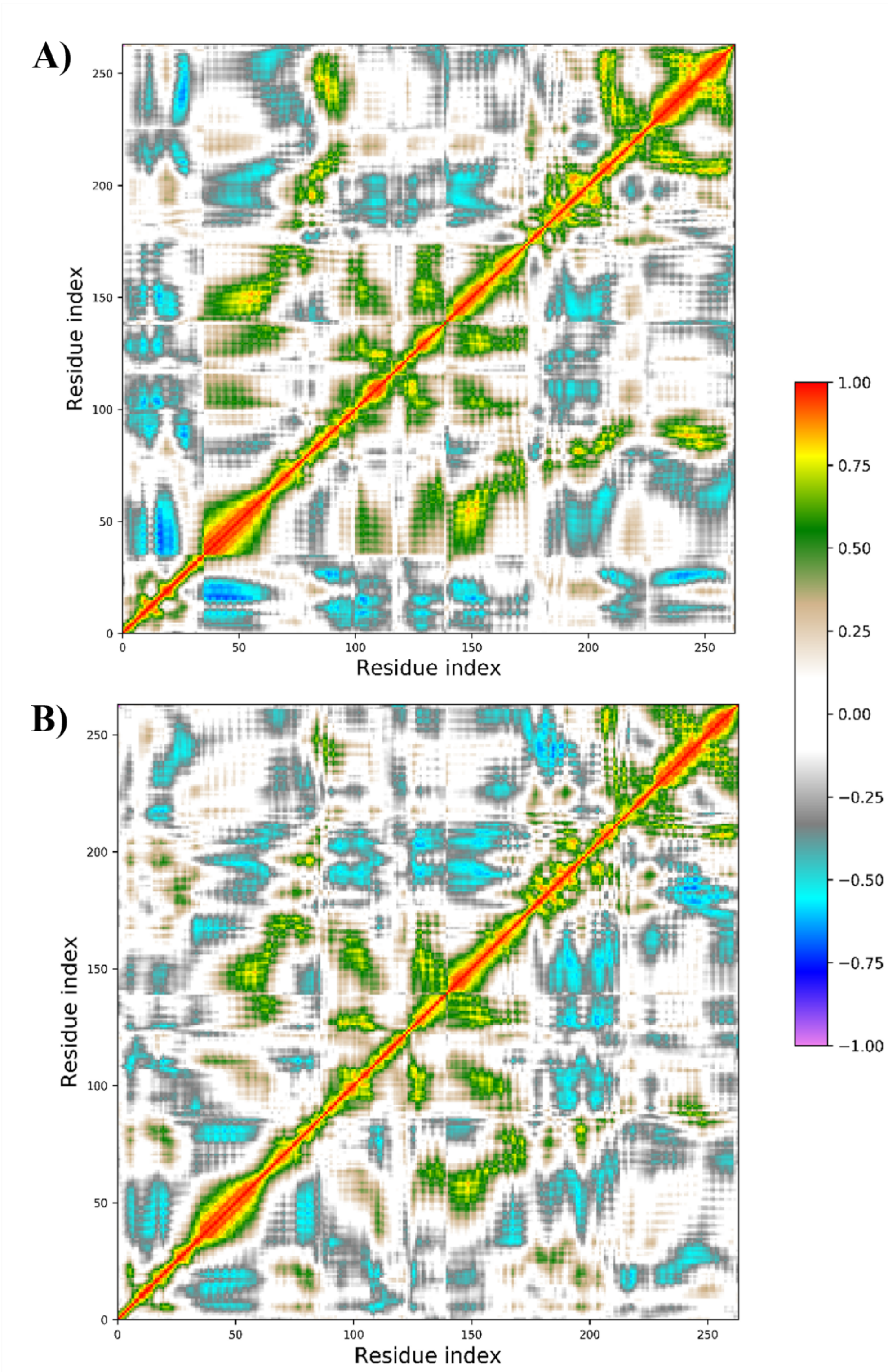

Figure S5. Dynamic cross-correlation maps calculated on the protein Cα carbons in absence (**a**) and presence (**b**) of the TTO molecules.

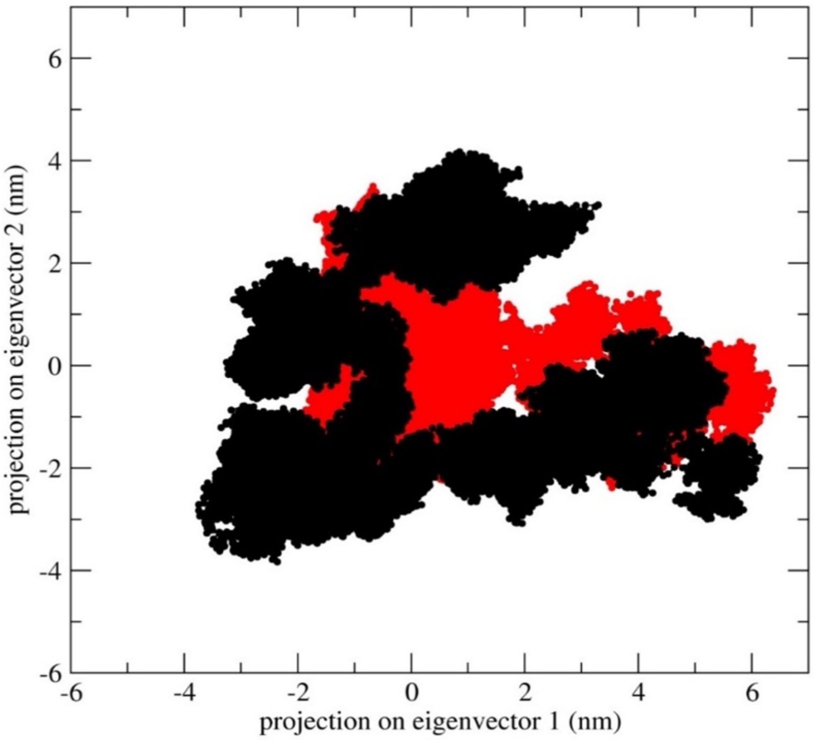

Figure S6. 2D projection of the motion identified along the first and second eigenvectors. The black colour identifies the conformational space sampled by the protein simulated in the absence of TTO, while the red one the space sampled in presence of the TTO compounds.

TTO molecule	Membrane component	Contact persistance (% simulation time)
terpinen-4-ol	DPPG-134	0.79
terpinen-4-ol	TMCL-139	0.68
terpinen-4-ol	TMCL-181	0.29
terpinen-4-ol	DPPG-158	0.16
1,8-cineole	TMCL-154	0.65
1,8-cineole	TMCL-120	0.44
1,8-cineole	DPPG-134	0.34
1,8-cineole	DPPG-158	0.10
1,8-cineole	TMCL-151	0.09
γ-terpinene	DPPG-251	0.60
γ-terpinene	TMCL-212	0.46
γ-terpinene	TMCL-255	0.39
γ-terpinene	DPPG-233	0.32
γ-terpinene	DPPG-215	0.27

Table S1. Contact analysis between the TTO molecules and the bacterial lipid membrane.

Salt Bridge	Salt bridge persistence (% simulation time)	
	System without TTO	System with TTO
Asp119-Arg120	51.0	88.0
Asp121-Arg111	20.0	7.0
Asp121-Lys242	34.5	10.5
Asp141-Arg146	63.8	100.0
Asp178-Lys216	/	95.0
Asp62-Lys149	/	100.0
Glu150-Arg142	18.0	92.5
Glu150-Lys147	25.0	100.0
Glu165-Arg68	97.0	70.0
Glu241-Arg263	27.0	43.0
Glu241-Lys244	49.0	39.0
Glu66-Arg61	/	40.0
Glu96-Arg97	75.0	93.0
Asp105-Lys107	87.5	/
Asp 121-Arg120	13.8	/
Asp 139-Lys134	18.0	/
Asp 141-Arg142	72.5	/
Glu 241-Arg120	26.0	/

Table S2. Salt bridges analysis for the two peptidoglycan glycosyltransferase systems. Salt bridges persistence is expressed as the percentage of simulation time in which the salt bridge is present in the protein. Salt bridges were taken into consideration up to a distance of 8.0 E with a percentage of persistence varying at least 10% between the two systems.

Hydrogen bond (sc: side chain; mc: main chain)	Hydrogen bond persistence (% simulation time)	
	System without TTO	System with TTO
Lys147/sc- Ser126/sc	13.01	0,60
Ala219/mc - Ser215/mc	16.68	0,86
Glu94/mc - Phe90/mc	29.77	11,41
Thr127/mc - Gln130/sc	0.29	20,78
Arg235/sc - Ser215/sc	0.68	15,53
Arg235/sc - Glu94/sc	4.46	26,63
Lys149/sc - Asp62/sc	0.12	48,18
Lys149/mc - Thr145/mc	4.87	33,68
Lys147/sc – Gln130/mc	41.84	/
Asn218/sc - Glu94/sc	41.25	19,27
Gly103/mc - Glu158/sc	37.55	54,87
Hsd5/sc - Hsd6/mc	10.22	/
Tyr136/sc - Glu66/sc	4.40	15,15
Arg97/sc - Asp105/sc	32.56	100,00
Arg68/sc - Glu71/sc	83.59	97,91
Tyr190/sc - Asp178/sc	34.41	46,76
Lys147/mc - Ser143/mc	13.30	24,46
Arg142/sc - Asp141/sc	10.25	/
Thr110/sc - Leu106-Mc	63.12	18,66
Leu213/mc - Gln209/mc	18.12	33,18
Tyr56/mc - Ile52/mc	13.92	25,91
Asn239/sc - Ala89/mc	26.69	13,25
Lys163/sc - Phe98/mc	17.44	0,41
Tyr136/sc - Asn63/mc	13.48	/
Leu151/mc - Lys147/mc	20.52	2,86
Arg120/sc - Glu241/sc	32.82	/
Tyr136/mc - Val132/mc	5.40	24,15
Lys242/sc - Glu96/sc	53.85	39,51
Lys107/sc - Asp105/sc	27.60	/
Lys87/sc - Glu84/sc	29.48	16,91
Asn188/mc - Glu184/mc	45.80	34,10
Tyr248/mc - Met243/mc	30.04	19,98
Arg156/sc - Asp62/mc	8.75	27,16
Met243/mc - Asn239/mc	38.11	28,03
Gln130/sc - Glu150/sc	36.91	/
Thr60/sc - Tyr56/mc	36.26	53,29
Gln131/sc - Ser126/mc	2.85	16,23
Thr129/sc - Glu158/sc	14.65	53,12
Tyr99/sc - Asn164/sc	20.61	/
Ser78-/sc - Asp80/sc	11.74	35,25
Ys29/mc - Lys26/sc	0.06	10,36
Lys163/sc - Glu158/sc	8.28	30,29
Tyr170/sc - Gln131/sc	11.22	40,77
Leu171/mc - Leu167/mc	22.49	12,52
Ser215/mc - Ala211/mc	23.87	4,63
Ile54/mc - Leu50/mc	31.42	45,48

Gln260/mc - Gln256/mc	15.62	/
Hsd10/sc - Val15/mc	13.95	/
Lys163/sc - Hsd101/mc	19.73	50,74
Val157/mc - Val153/mc	9.31	27,86
Arg17/sc - Gly18/mc	20.14	/
Thr116/mc - Asp121/sc	20.10	/
Thr116/sc - Arg111/mc	11.89	/
Arg235/sc- Asn231/sc	16.47	/
Gln253/mc - Asn250/sc	21.79	50,09
Ile46/mc - Leu42/mc	19.41	33,98
Ile40/mc - Ile36/mc	24.78	10,23
Hsd102/sc - Glu158/sc	9.25	32,50
Thr116/sc - Ser115/mc	11.99	/
Lys134/sc - Asp139/sc	15.65	/
Ser11/mc - Val15/mc	14.30	/
Arg68/sc - Glu165/sc	15.12	54.18
Ser115/sc - Leu113/sc	17.10	/
Lys69/sc - Glu66/sc	43.31	18.16
Thr182/sc - Ser75/mc	12.45	1.08
Met55/mc - Phe51/mc	31.06	14.16
Lys147/sc - Glu150/mc	69.20	2.24
Arg142/sc - Glu150-Lc	21.87	66.44
Ser237/sc - Thr233/mc	48.59	61.57
Val153/mc - Lys149/mc	11.51	48.45
Arg146/sc - Asp141/mc	10.28	/
Lys13/sc - Glu150/sc	21.23	/
Arg235/sc - Asn218/sc	14.89	0.43
Thr110/mc - Leu106/mc	24.52	2.51

Table S3. Hydrogen bond analysis for the two systems considering variation of at least 10% between the two systems.

System components	System without TTO	System with TTO
DPPG	34686	34686
TMCL1	50160	50160
Ergosterol	3540	3540
Stigmasterol	2340	2340
Terpinen-4-ol	0	319
1,8-cineole	0	116
γ-terpinene	0	78
Na^+ ions	865	916
Cl^- ions	353	404
Water molecules	374295	428160
Total atoms	470450	524930

Table S4. Atomic composition of the two peptidoglycan glycosyltransferase simulations systems
